# Inclusion of Ethnic Minorities in Telehealth Trials for Type 2 Diabetes: Protocol for a Systematic Review Examining Prevalence and Language Issues

**DOI:** 10.2196/resprot.5195

**Published:** 2016-03-11

**Authors:** Louisa Edwards, Leila Rooshenas, Talia Isaacs

**Affiliations:** ^1^ University of Bristol Graduate School of Education Bristol United Kingdom; ^2^ University of Bristol School of Social and Community Medicine Bristol United Kingdom

**Keywords:** telemedicine, telehealth, type 2 diabetes, diabetes mellitus, ethnic minorities, trial recruitment, external validity, systematic review, English proficiency, health communication

## Abstract

**Background:**

Type 2 diabetes is common, on the rise, and disproportionately affects ethnic minority groups. Telehealth interventions may mitigate diabetes-related complications, but might under-recruit or even exclude ethnic minorities, in part because of English language requirements. The under-representation of minority patients in trials could threaten the generalizability of the findings, whereby the patients who might stand to benefit most from such interventions are not being included in their evaluation.

**Objective:**

The aims of this systematic review are twofold: (1) to assess the reporting and prevalence of ethnic minorities in published telehealth trials for type 2 diabetes, including identifying trial features associated with successful patient recruitment; and (2) to determine the proportion of such trials that report English language proficiency as an inclusion/exclusion criterion, including how and why they do so.

**Methods:**

Randomized controlled trials (RCTs) of adults with type 2 diabetes in Western, English-speaking countries that included telehealth interventions targeting diabetes as a primary condition, and those that did not specifically recruit minority groups will be included. Search strategies were devised for indexed and keyword terms capturing type 2 diabetes, telehealth/health technology, and RCTs in English language publications from 2000 to July 2015 in MEDLINE, PsycINFO, EMBASE, CINAHL, and CENTRAL. Reference lists of included studies will also be searched. Two reviewers will independently screen abstracts and full-text articles against inclusion criteria, mediated by a third reviewer if consensus cannot be reached. Data extracted from included studies will be checked by a second reviewer and will be summarized using narrative synthesis.

**Results:**

This research is in progress, with findings expected by Spring 2016.

**Conclusions:**

This review will address research reporting and recruitment practices of ethnic minorities in telehealth RCTs for type 2 diabetes. Prevalence estimates will elucidate generalizability of existing research, with implications for researchers, health professionals, and policy makers. Identifying trial or intervention features that appear to facilitate ethnic minority recruitment, as well as language barriers that impede it might suggest ways to improve recruitment in future trials.

**Trial Registration:**

PROSPERO International Prospective Register of Systematic Reviews: CRD42015024899; http://www.crd.york.ac.uk/PROSPERO/display_record.asp?ID=CRD42015024899 (Archived by WebCite at http://www.webcitation.org/6fUMqbJ0f).

## Introduction

Diabetes is a common chronic condition, with global estimates of 387 million diabetic adults worldwide in 2014 [1]. This figure is projected to rise rapidly in the coming years [1], which will further increase the financial burden on health care systems. About 90% of diabetic patients have type 2 diabetes, which typically affects those of middle age or older, although the growing incidence of childhood obesity means that this condition is now occurring in younger populations as well [1]. Risk of developing type 2 diabetes can be reduced by making healthier lifestyle changes, while subsequent type 2 diabetes-related complications, such as heart disease and stroke, can be reduced from good diabetes management [2,3]. Therefore, many behavioral interventions, including those making use of telehealth, which is defined here as the use of technology to support the remote delivery of health care and promote self-management [4], have targeted this condition.

Telehealth could help health systems to cope with the increasing pressures they face with an aging population experiencing rising rates of chronic conditions, and also empower patients to self-manage their care. A key benefit of telehealth is that it can improve access to care for those who experience difficulties utilizing traditional health care [5]. Indeed, telehealth is attracting international interest as an alternative to traditional face-to-face care [6]. While the effectiveness evidence of telehealth from randomized controlled trials (RCTs) is mixed and might vary by chronic condition, systematic reviews of telehealth interventions for type 2 diabetes have generally shown beneficial clinical effects, such as improved blood glucose levels [7-9]. However, many telehealth trials conducted in Western countries recruit mostly white patients [10-12] or may not report the ethnicity of participants [13].

The under-recruitment of certain sociodemographic groups, such as ethnic minority populations, to telehealth RCTs jeopardizes the generalizability of trial findings. This issue is particularly pressing in the case of type 2 diabetes, because the condition is about six times more prevalent in South Asians and up to three times more common in those of African and Afro-Caribbean descent [14]. Across ethnic minorities, the prevalence of type 2 diabetes is 5.7%, whereas it is 1.7% for non-Hispanic white ethnic groups [1]. If the effectiveness and acceptability of telehealth interventions for type 2 diabetes are mostly being trialed among white patients, whether the same interventions would be equally beneficial in ethnic minority groups—those with comparatively greater health needs in this regard—is not being adequately addressed. This has implications when considering new service delivery deployment of such telehealth interventions.

There is some evidence for the low levels of ethnic minority participation in telehealth interventions for diabetes, despite the fact that ethnicity is not widely reported. In a systematic review of 26 studies of interactive computer-based interventions for diabetes, only 8 of these studies reported on the inclusion of ethnic minorities, whereby the median proportion of ethnic minority participation was 39% of the recruited study sample (within-study range of ethnic minority participants: 5-100%) [15]. Further to this initial investigation, a secondary aim of a recent review by Cotter et al [16] was to examine the degree to which Internet interventions were tailored to diverse or underserved diabetic populations. The ethnic mix of participants was reported in 4 of the 9 studies included in this review, whereby there was between 24% and 100% minority group participants across these 4 studies. Another review published in the same year (2014) reported that half of the 16 included RCTs contained minority prevalence information, in which the within-study range of ethnic minority participants was 15-100% [9].

While these reviews provide some indication about the lack of minority patient participation and the under-reporting of ethnicities in telehealth research for diabetes, they are subject to several limitations. These consist of including both RCTs and other study designs within the review [15,16], including studies of patients with type 1 and type 2 diabetes [9,15], and only considering a narrow range of computer technologies, to the exclusion of other telehealth interventions [15,16]. RCTs are held as the “gold standard” in health research because they reduce bias and potential confounding, but they may attract participants who differ from those taking part in other types of studies, due to the time commitment required for participation and other factors [17,18]. Furthermore, since it is only type 2 diabetes that disproportionately affects ethnic minority groups, rather than type 1 diabetes, it is more important to establish the prevalence of ethnic minorities in studies that are restricted to type 2 diabetic patients. In addition, excluding studies that make use of glucose monitoring or that are telephone based means that a large volume of telehealth research in this area was not considered. Finally, a fundamental issue affecting prevalence estimates of ethnic minorities in the 2 more recent reviews [9,16]—albeit not the focus of either review—is that they included studies that, during recruitment, specifically targeted just one or more ethnic minority groups. This is problematic because it overestimates and biases prevalence estimates, as well as masks overall user acceptance in the general patient population. To overcome these limitations, a more comprehensive systematic review of the literature is required, as well as exploring potential barriers and facilitators to ethnic minority participation in telehealth trials.

One frequently cited challenge in reference to minority group participation in RCTs relates to patients’ language proficiency and literacy [19,20]. Among those living in the United States, around 25 million people are unable to speak English fluently [21], while it is estimated that almost 300,000 adults in England and Wales from the 4 most common ethnic minority groups speak little or no English [22]. Ensuring that patients have the requisite language ability to understand the conditions of research participation is essential in all research studies for ethical reasons (eg, obtaining informed consent). Moreover, because a fundamental component of telehealth interventions involves non-face-to-face communication, being able to engage in efficient communication is integral to participating in and benefiting from telehealth interventions. It is unclear whether researchers are utilizing objective and systematic ways of assessing whether patients have the necessary language skills to participate in telehealth RCTs for diabetes, or whether such decisions are made more subjectively. The role that language plays in telehealth trial participant inclusion or exclusion decisions, therefore, needs to be systematically investigated. This is of particular significance for type 2 diabetes trials, where minority patients who potentially stand to benefit most from such interventions need to be proportionally represented, and language barriers may impede their ability to do so in telehealth trials targeting this condition. To our knowledge, no systematic review has examined language as a potential barrier to ethnic minority participation in telehealth trials for type 2 diabetes.

Taken together, the potential under-recruitment of ethnic minorities and language barriers present two major issues facing telehealth RCTs for type 2 diabetes. Both affect the generalizability of trial findings and have wider implications for the adoption of new telehealth services into health care systems, as well as equitable access to health care. Therefore, the aims of this systematic review are twofold: (1) to assess the reporting and prevalence of ethnic minorities in published telehealth trials for type 2 diabetes, including identifying trial features associated with successful patient recruitment (trial includes ≥30% ethnic minorities, in line with median prevalence across earlier reviews [9,15,16]); and (2) to determine the proportion of such trials that report English language proficiency as an inclusion/exclusion criterion, including how and why they do so. Building on the previously mentioned reviews [9,15,16], our study will update and refine the ethnic minority prevalence figures with more stringent criteria (ie, RCTs only, adult type 2 diabetes patients only, excluding studies with an ethnically targeted sample) intended to minimize the effects of extraneous variables that could bias estimates. Compared with the earlier reviews, we will also include a broader range of telehealth interventions that make use of any technology medium. In addition, we will move beyond simple reporting and seek to identify the characteristics of trials that have higher proportions of ethnic minority participation.

## Methods

This systematic review will be conducted in accordance with the *Cochrane Handbook for Systematic Reviews of Interventions* [23] and will follow the Preferred Reporting Items for Systematic Reviews and Meta-Analyses (PRISMA) guidelines [24].

### Search Strategy

We will search for potentially eligible studies using MEDLINE (via Ovid), PsycINFO (via Ovid), EMBASE (via Ovid), CINAHL (via EBSCOhost), and the Cochrane Central Register of Controlled Trials (CENTRAL, via Wiley Online Library) from January 1, 2000, to July 31, 2015. Reference lists of included studies will also be searched.

Both Medical Subject Heading (MeSH) terms and keywords will be used in the searches to capture themes of type 2 diabetes, telehealth/health technology, and RCTs (see Multimedia Appendix 1 for MEDLINE example). Because of the varied terms researchers tend to use to describe telehealth, the search incorporates numerous plausible key terms. We also examined key terms used in related diabetes telehealth systematic reviews with similar inclusion and exclusion criteria to ensure comprehensive searching [7-9,25]. We consulted a medical subject librarian to finalize the strategy. Search filters restrict results to English language publications, humans, and the relevant publication dates.

### Screening and Study Selection

The database searches will be performed by one reviewer (LE). Citations and abstracts will be uploaded into EndNote X7, de-duplicated, and screened for eligibility against the inclusion criteria by the same reviewer (LE). A second reviewer (KB) will concurrently and independently assess the titles and abstracts against the inclusion criteria. Discrepancies will be resolved through discussion and achieving consensus, or by consulting a third reviewer (TI) if consensus cannot be reached. Reasons for excluding abstracts will be recorded for later reporting.

After retrieving the full text of the studies that remain after the first stage of assessment, 2 reviewers (LE and DW) will independently review the full text for further eligibility assessment. Reasons for exclusion will be recorded for reporting in the PRISMA flow diagram. As before, discrepancies will be resolved through consensus or by consulting a third reviewer (TI) when consensus cannot be reached. Multiple reports from the same study will be linked for included studies.

### Inclusion Criteria

The following criteria will be used to select studies for inclusion in the review.

#### Study Designs

Only RCTs will be included, which could include two- or more arm trials, pilot studies, cross-over designs, cluster RCTs, and so on.

#### Participants

The review will be limited to adult patients (aged ≥ 18 years) of either sex with type 2 diabetes in Western countries where English is both an official and a majority language (USA, Canada, the United Kingdom, Ireland, Australia, and New Zealand). Only participants recruited widely from the general patient population will be included, rather than RCTs that specifically aim to include just one or more ethnic groups. This is because we are interested in assessing the prevalence of ethnic minorities in typical telehealth RCTs, in which the focus is simply on achieving the target sample size. Studies that include a homogeneous ethnic minority sample will artificially inflate the prevalence results. However, trials recruiting from other specific contexts (eg, urban/rural; economically deprived/low-income/medically underserved groups) will be permitted. We acknowledge that there may be a relationship between ethnicity and these other sociodemographic characteristics, but studies that include such samples are unlikely to result in completely homogeneous ethnic samples. In addition, telehealth is meant to address barriers to accessing traditional health care, and, therefore, recruitment within these contexts is a direct test of a typically cited advantage of telehealth [26].

#### Interventions

Telehealth interventions of any duration, using any technology medium specifically designed to treat or improve type 2 diabetes as the primary condition, will be included. By this, we mean interventions targeting or monitoring blood glucose levels, diabetes education/knowledge, medication adherence, and diabetes self-care behaviors (eg, foot checking and exercise/diet/weight interventions), whereas mental health interventions and treatment of other secondary diabetes-related complications (eg, foot ulcers, cardiovascular health) will not be included. As outlined in the “Introduction” section and illustrated in the search strategy (see Multimedia Appendix 1), we take a broad definition of telehealth, which includes telemedicine, telemonitoring, teleconsultations, medical informatics, eHealth and mHealth (electronic or mobile health), or other forms of remote health care delivery, treatment, and support. However, the intervention must be described by the authors as telehealth or the majority of the intervention must be delivered electronically, rather than in-person.

#### Comparators

We will include studies employing any comparison group within an RCT, such as usual care, wait-list control group, or head-to-head trials.

#### Outcomes

Studies will not be selected on the basis of any reported outcomes. The primary outcomes of the review will include

1. Proportion of RCTs that report on the ethnic/racial composition of trial participants.

2. Overall prevalence of ethnic minorities reported in telehealth trials for patients with type 2 diabetes (between-study median and range).

3. Features of studies that include a higher proportion of ethnic minorities (≥30% of the sample), such as country, recruitment setting (primary care, community, secondary care), telehealth medium (telephone, video, email, etc), resource availability (translation/interpretation), targeting of low-income recipients, tailoring of intervention (to individual needs, cultural group, etc).

4. Proportion of telehealth trials for type 2 diabetes that report English language proficiency (oral/written) or literacy as inclusion/exclusion criteria.

5. Way in which English language proficiency/literacy is operationalized by authors reporting this as inclusion/exclusion criteria.

6. Reason(s) that patients are excluded on language grounds (eg, ethical considerations, ability to participate in intervention, lack of translation/interpretation resources).

#### Setting and Timing

There are no restrictions on the study setting or length of intervention or follow-up.

#### Report Characteristics

The review will include RCTs published in English in peer-reviewed journals between 2000 and 2015. The rationale for this 15-year publication restriction is that we are interested in capturing the current state of telehealth RCTs for type 2 diabetes. Health technology has evolved rapidly within this period and telehealth trials have also proliferated in recent years, and so this time frame should provide an up-to-date and sufficient summary of ethnic minority participation and language recruitment barriers in trials.

### Exclusion Criteria

Because of the greater risk of bias and potential confounds, study designs other than RCTs (eg, observational studies, surveys), including nonrandomized controlled studies, will be excluded. The following additional exclusions apply: (1) systematic or other literature reviews, letters, commentaries/editorials, study protocols, conference abstracts, or presentations; (2) secondary subgroup analyses of RCTs or RCT-generated data modeling studies; (3) RCTs not published in peer-reviewed journals, such as dissertations and case reports; (4) targeted ethnic minority trials (could be a single ethnic minority sample, or a dedicated comparison between one or more ethnic groups), although the number of such studies and recruited patients of various ethnic backgrounds that would otherwise have met our eligibility criteria will be recorded; (5) mixed (type 1 and type 2) diabetes samples, type 1 diabetes, gestational (pregnancy) diabetes, or diabetes insipidus; (6) studies involving adolescents or children; (7) telehealth interventions directed at and solely experienced by health professionals, even if the study measures the effect of such interventions on type 2 diabetic patients; and (8) telehealth interventions directed solely at diabetes-related complications, such as diabetic retinopathy, hypertension, diabetes distress. Interventions addressing both primary and secondary diabetes issues will, however, be included.

### Data Collection

Data extraction forms will be developed for the current review using existing guidelines [23]. The form will contain information about the (1) study details (eg, country, recruitment setting, design, inclusion and exclusion criteria), (2) participant demographics (eg, sample size, age, sex, ethnic mix, baseline diabetes severity), and (3) intervention characteristics (eg, description, duration, frequency, telehealth medium). A complete list of all variables is available through PROSPERO [27]. Outcome data will be abstracted by one reviewer (DH) into Excel, and independently checked by a second reviewer (DW). Disagreements will be resolved by a third reviewer (TI).

The Cochrane risk of bias tool [28] will be used to assess selection, performance, detection, attrition, reporting, and “other” sources of bias. Accordingly, a judgment of “high,” “low,” or “unclear” bias along with supporting justification will be provided and recorded using Review Manager (RevMan, version 5.3; Copenhagen: The Nordic Cochrane Centre, The Cochrane Collaboration, 2014). Two reviewers (LE and DH) will assess all studies, which will be checked by another reviewer (DW). As before, disagreements will be resolved through discussion or by consulting a third reviewer (TI).

We anticipate a high degree of missing or unreported ethnicity data in the studies, as well as few details regarding English language inclusion or exclusion criteria. It is, however, beyond the scope of this review to supplement recruitment-related information provided in refereed journal articles by consulting the gray literature or contacting authors for missing information. While we acknowledge this limitation, this is consistent with the overall goal of the review to systematically document what is and is not reported in peer-reviewed trial publications.

### Data Synthesis

Proportions of ethnic minorities included across studies (overall, and by specific ethnic group), as well as frequencies of trials reporting English language as inclusion/exclusion criteria, will be presented for individual included studies and aggregated across included studies.

The results will be presented in a narrative synthesis using the synthesis framework developed by Popay and colleagues [29]. The framework sets out the following 4 key elements to narrative synthesis: (1) developing a theory of how the intervention works, why, and for whom; (2) developing a preliminary synthesis of findings of included studies; (3) exploring relationships in the data; and (4) assessing the robustness of the synthesis. In terms of the first element, which focuses on constructing a theory around the intervention’s effectiveness, we will re-frame this according to our final research question concerning what features of telehealth trials for type 2 diabetes tend to successfully recruit a sizeable proportion of ethnic groups. We are interested in the kinds of recruitment techniques, studies, or interventions that appear to result in more ethnically balanced telehealth trial samples (≥30% ethnic minority participants). These insights could inform current recruitment practice for targeting ethnically diverse patients in telehealth studies, taking language support and other factors into account. Thus, we will seek to develop a theory around this facet. We will use an inductive approach, in which a theory will be formulated based on themes or patterns that emerge from the data.

As outlined in Popay and colleagues [29], several tools and techniques will be used in processing the data around each of the elements, which could include textual descriptions, groupings and clusters (eg, by telehealth intervention medium, country), tabulation, thematic analysis, concept mapping, and reflecting critically on the synthesis process. In line with the guidance, we will undertake these processes iteratively and will discuss the emerging results with the research team throughout.

An additional, retrospective subgroup analysis (added after PROSPERO registration) will be carried out on trials that specifically targeted ethnic minority groups as part of their recruitment strategy. The aim of this analysis will be to identify trial features or strategies that were employed that potentially resulted in heightened recruitment of these targeted groups. All such trials will have otherwise met the inclusion criteria for the full systematic review, except that recruitment was aimed at one or more ethnic minority groups.

## Results

This systematic review is currently underway, with results anticipated by Spring 2016.

## Discussion

This systematic review will provide up-to-date prevalence estimates of research-reporting practices and participation rates of ethnic minorities in telehealth trials for type 2 diabetes that make use of a broad range of technologies. The inclusion of ethnic minorities in type 2 diabetes telehealth research is methodologically important to maximize the external validity of findings, in addition to extending the potential benefits of telehealth type 2 diabetes research to a wider cross section of patients. These factors, coupled with the rapid rise of telehealth interventions, make the need to assess language as a potential recruitment barrier for minority participation, as well as highlighting facilitators to recruitment, all the more pressing. Moreover, determining which kinds of trials or interventions tend to attract a higher proportion of ethnic minorities has significance for a variety of stakeholders in the health care system. This could inform trial recruitment strategies, thereby enhancing the ethnic mix within trials, increasing the external validity of findings, while also ensuring a broad spectrum of diabetic patients evaluate and potentially benefit from such telehealth interventions. From a more macro level, greater insight into the characteristics of trials that successfully recruit a heterogeneous population is synchronous with the overarching goal of promoting greater social inclusiveness and more equitable access to health care.

Ethnic minority participation in type 2 diabetes telehealth RCTs will impact health systems when considering commissioning new services for patients, in which effectiveness across ethnically diverse groups and, in particular, among ethnic minorities that are disproportionately affected by type 2 diabetes must be demonstrated. While the 3 previously cited reviews [9,15,16] marked an important first step in tabulating the prevalence of ethnic minorities included in telehealth interventions for diabetes, they also highlighted how few studies (31-50%) actually reported on the ethnic mix of the study sample. If this is still the case in this up-to-date comprehensive review, despite the widespread adoption of best reporting practices for studies, such as the Consolidated Standards of Reporting Trials 2010 (CONSORT) statement [30], then this suggests the need for further attention in research reporting. These guidelines state that baseline characteristics of trial participants should be reported, which is predicated by the fact that this information is gathered as part of data collection. We contend that this is especially pressing in trials of chronic conditions in which there is known to be variation in prevalence by sociodemographic variables, and that it is precisely these variables that constitute which key baseline characteristics ought to be reported.

## Appendix

Multimedia Appendix 1 MEDLINE search strategy using Ovid interface.

## Abbreviations

CONSORT: Consolidated Standards of Reporting Trials
MeSH: Medical Subject Heading
PRISMA: Preferred Reporting Items for Systematic Reviews and Meta-Analyses
RCTs: randomized controlled trials
