# Supplementary material for: Inclusion of Ethnic Minorities in Telehealth Trials for Type 2 Diabetes: Protocol for a Systematic Review Examining Prevalence and Language Issues
Source: JMIR Res Protoc. 2016 Mar 11;5(1):e43. doi: 10.2196/resprot.5195 (PMC4808238; doi:10.2196/resprot.5195)
Supplement: Multimedia Appendix 1 [file resprot_v5i1e43_app1.pdf]

MEDLINE (via Ovid) search strategy

1. exp Diabetes Mellitus, Type 2/
2. diabet$.tw
3. (non insulin$ depend$ or noninsulin$ depend$ or non insulin?depend$ or noninsulin?depend$).tw
4. (adult?onset diabet$ or adult onset diabet$).tw
5. type 2 diabet$.tw
6. or/1-5
7. exp Computers/
8. exp Internet/
9. exp Electronic Mail/
10. video$.tw
11. (world wide web or worldwide web or website$ or web$).tw
12. exp Telephone/
13. exp Cell Phones/
14. exp Telemedicine/
15. exp Medical Informatics/
16. exp Remote Consultation/
17. telediabete$.tw
18. (telehealth or tele?health or tele health).tw
19. (telehealthcare or tele?healthcare or tele?health care or tele health care or tele healthcare).tw
20. (telemonitor$ or tele?monitor$ or tele monitor$).tw
21. (e?health or ehealth).tw
22. electronic health$.tw
23. (m?health or mhealth).tw
24. mobile health$.tw
25. ((computer-assist$ or computer-based or web-based or telephone-based) adj6 (therap* or treatment* or support* or education*)).tw
26. (computer$ or Internet).tw

27. (interactive or online or on-line or telemedicine$ or video$ or cellular phon$ or mobil$ phon$).tw

28. or/7-27

29. 6 and 28

30. randomi?ed.tw

31. exp Randomized Controlled Trials as Topic/

32. exp Controlled Clinical Trial/

33. (randomi?ed) adj3 (control$) adj3 (trial).tw

34. (control$ or clinical) adj3 (trial).tw

35. (random$ adj6 (allocate$ or assign$ or basis or order$)).ab,ti.

36. or/30-35

37. 29 and 36

38. (comment or editorial or historical-article or meta-analysis or review).pt

39. 37 not 38

40. Diabetes Mellitus, Type 1/

41. Diabetes insipidus/

42. Gestational diabetes/

43. or/40-42

44. 39 not 43

45. limit 44 to (english language and human and yr="2000 – Current")

46. remove duplicates from 45
